# How Far Are We from Visceral Leishmaniasis Elimination in Bangladesh? An Assessment of Epidemiological Surveillance Data

**DOI:** 10.1371/journal.pntd.0003020

**Published:** 2014-08-21

**Authors:** Rajib Chowdhury, Dinesh Mondal, Vashkar Chowdhury, Shyla Faria, Jorge Alvar, Shah Golam Nabi, Marleen Boelaert, Aditya Prasad Dash

**Affiliations:** 1 National Institute of Preventive and Social Medicine (NIPSOM), Mohakhali, Dhaka, Bangladesh; 2 ICDDR,B, Mohakhali, Dhaka, Bangladesh; 3 Dhaka College, New Market, Dhaka, Bangladesh; 4 DNDi, Geneva, Switzerland; 5 Directorate General of Health Services (DGHS), Mohakhali, Dhaka, Bangladesh; 6 Institute of Tropical Medicine, Antwerp, Belgium; 7 National Institute for Malaria Research, New Delhi, India; New York University, United States of America

## Abstract

**Introduction:**

In 2005, Bangladesh, India, and Nepal joined forces to eliminate Visceral Leishmaniasis (or kala-azar) from the region by 2015. In Bangladesh the elimination target is set at less than one new case per 10,000 population per year at upazila (sub-district) level. As the deadline approaches, we review the status of the elimination initiative in this country.

**Methods:**

We collected all available disease surveillance data at the Disease Control Unit of the Directorate General of Health Services, Government of Bangladesh from 1994 to 2013. Additionally, we retrieved data from the Civil Surgeon Office from the Mymensingh district, one of the most heavily affected areas in Bangladesh.

**Results:**

Between 1994 and 2013, 109,266 kala-azar cases causing 329 deaths were reported from 37 endemic districts in Bangladesh. Only 16 districts reported cases every year. The Mymensingh district was the most affected with 53,582 (49.04%) cases. Between 2008 and 2013 only 16 upazilas showed incidence rates above the elimination target in which they ranged from 1.06 to 18.25 per 10,000 people per year.

**Discussion:**

While clear progress has been made towards eliminating VL, 16 upazilas in Bangladesh had not yet reached the target in 2013, based on official notification data that probably suffered from under-reporting bias. The elimination initiative urgently needs to establish methods to ascertain and monitor the elimination target.

## Introduction

On the Indian subcontinent, Visceral Leishmaniasis (VL), or kala-azar, is caused by *Leishmania donovani*, which is transmitted from man to man by the sand fly *Phlebotomus argentipes*, the only known vector [1]. Of the 200,000 to 400,000 new cases of VL worldwide, more than 90% are reported from India, Bangladesh, Sudan, South Sudan, Ethiopia, and Brazil [2]. VL affects the poorest communities in these countries and is almost always fatal if not treated. The first report of VL ever came from Jessore, currently located in southwestern Bangladesh, where an epidemic outbreak killed an estimated 75,000 people between 1824 and 1827 [3]. Over the next decades, kala-azar became endemic in the region and spread slowly through Bengal, where it devastated the population of Burdwan and other areas. Subsequently, the disease spread eastward into Assam. Between 1892 and 1898, one-third of the population of the Nowgong district in Assam, India died [4]. Another epidemic of VL in conjunction with the Spanish Influenza epidemic claimed a further 200,000 lives in Assam and in the Brahmaputra valley between 1918 and 1923 [5]. Up to 1940, more than 1,000,000 VL cases were reported in former Bengal where the first mass treatment measures were undertaken [6], [7]. The incidence finally declined because of the dichlorodiphenyltrichloroethane (DDT) spraying by the Malaria Eradication Programs in the 1950s, and VL was thought to be eliminated by 1970. Between 1968 and 1980, only 59 cases were reported in Bangladesh [8]. But since the 1980s, after the interruption of DDT spraying, there has been a dramatic resurgence of VL, with 73,467 cases reported from Bangladesh between 1994 and 2004, and many more reported in India [3].

In 2005, Bangladesh, India, and Nepal joined efforts to eliminate kala-azar. Elimination was thought feasible in this region because (i) human beings are considered the only reservoir host of *L. donovani*, (ii) *Phlebotomus argentipes* is the only vector, (iii) the disease is confined to a limited number of districts, (iv) a rapid diagnostic test allows easy diagnosis, and (v) effective oral treatment was available [9]. The respective Health Ministers of the three countries signed a Memorandum of Understanding (MOU) with the aim to reduce the annual incidence rate of VL to less than one per 10,000 inhabitants in the endemic communities by 2015, an elimination goal endorsed by the World Health Organization (WHO) [9]. The five strategies adopted in the VL elimination initiative were (i) early diagnosis and treatment, (ii) strengthened epidemiological surveillance, (iii) integrated vector management, (iv) social mobilization, and (v) operational research [9].

For any disease control program, proper epidemiological surveillance is a key issue. It allows for the establishment of the past and present disease burden, and will guide the program to take timely and appropriate action on case detection, patient management, vector control, and community awareness. In an elimination initiative, routine surveillance data are essential to keep track of the elimination target, though additional measures are required to ascertain the elimination status of given areas. As the set VL elimination target of 2015 is fast approaching, we have analyzed the available epidemiological information in Bangladesh to advise the national and regional disease control policy.

## Methods

### Context

Bangladesh is administratively divided into six divisions, namely: Chittagong, Barishal, Dhaka, Khulna, Rajshahi, and Sylhet. All of the divisions except Sylhet are reporting VL cases, but not to the same extent. Each division is further subdivided into districts (a total number of 64), sub-districts, called upazilas or thanas (a total of 482), Union Parishads (UPs), and wards. The governmental health care system is structured along the same administrative divisions, with a national, divisional, district, upazila (sub-district), union, and ward level. Three levels in this system deliver VL treatment: (i) the Upazila Health Complex (UHC) (the lowest level, offering indoor facilities with 31 to 50 beds) (ii) the District Sadar Hospital (DSH) (a 200- to 500-bed hospital), and (iii) Medical Colleges. A UHC caters to a population of 200,000 to 300,000, while a DSH covers approximately 2 to 5 million inhabitants.

### Epidemiological surveillance system

Disease surveillance is organized as follows. At the end of each month, the UHC and DSH send their morbidity and mortality reports to the Civil Surgeon Office at the district headquarters. The District Civil Surgeon transmits these reports to the Director of Disease Control at the Directorate General of Health Services (DGHS), who will notify country data to the World Health Organization. As is commonly the case in routine surveillance, these figures are an underestimation of the true number of VL cases. Medical colleges do not report although some VL patients get reported when they are referred back to the UHC or DHS after some days of treatment. This happens quite often, as VL drugs are provided for free in the public health services but are not always available in the medical colleges. Another important factor to consider when analyzing trends in the surveillance data is the recent change in drug policy. While the injectable Sodium Stibogluconate (SSG) was the only drug of choice for many years, since the middle of 2009, the oral drug Miltefosin was introduced as first-line drug (except for women of childbearing age and pregnant women). As this drug is not available in the local market, patients are now more motivated to attend a UHC to get this oral medication. The recent introduction of single-dose AmBisome therapy also attracts VL patients to the UHC, as does the recent involvement of one international Non-governmental organization (NGO), Médecins Sans Frontières (MSF), in VL care.

### Data collection and analysis

We have collected all data on VL available at the central level (DGHS) for the years 1994 to 2013. We also collected district-level data for the Mymensingh district from the Civil Surgeon's Office in Mymensingh for the same period. As the VL elimination target is set at the upazila level “to reduce VL incidence rate below one per 10,000 population per year at upazila level,” [9] we calculated the Incidence Rates (IR) for each upazila. We used the total population of the upazila in the corresponding year in the denominator. The data were analyzed by using SPSS, Minitab, and Microsoft Excel.

## Results

**Figure 1:**
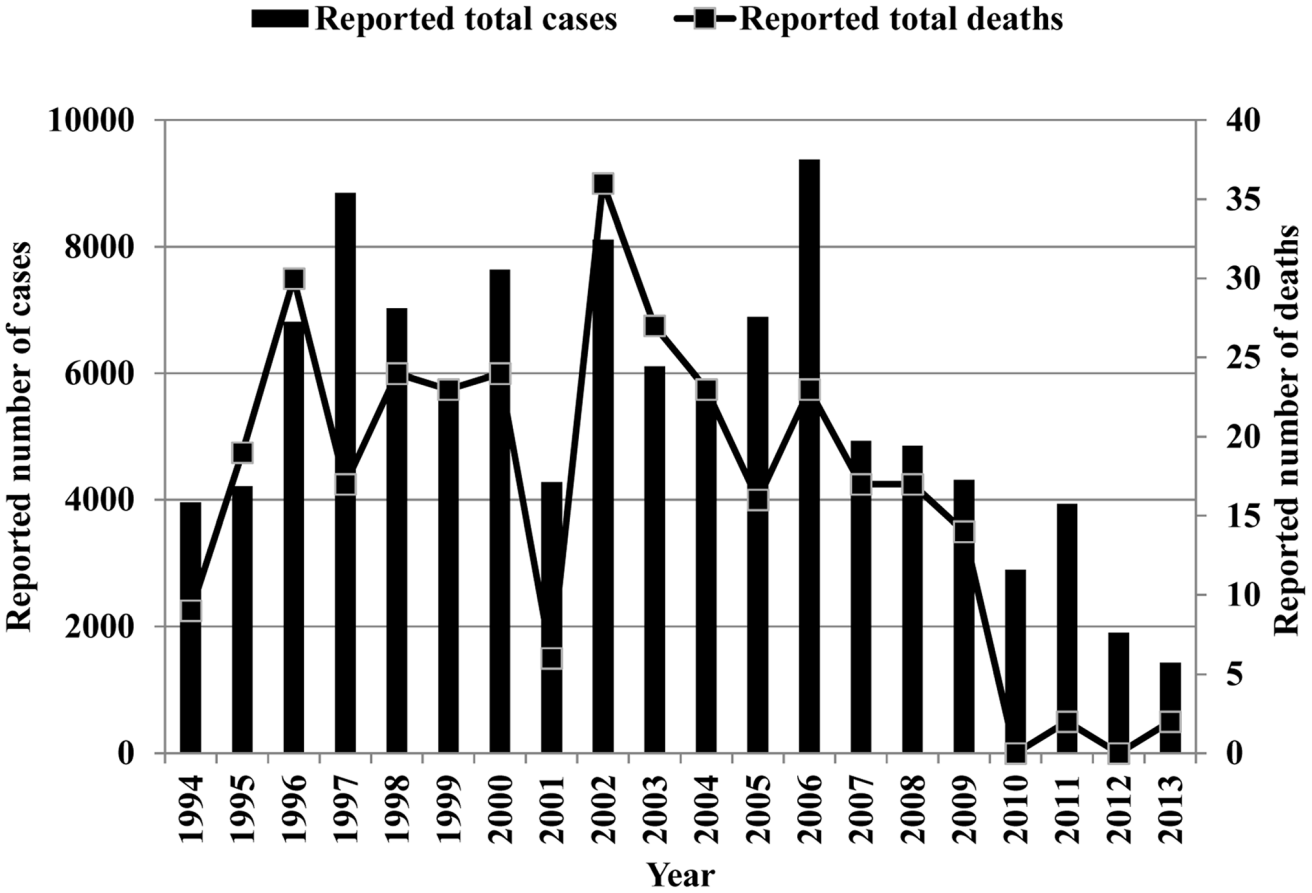
The total number of Visceral Leishmaniasis (VL) cases and deaths reported from 1994 to 2013 in Bangladesh. Source: Malaria and Vector-Borne Disease Control Unit, Directorate General of Health Services (DGHS), Dhaka, Government of Bangladesh.

**Figure 2:**
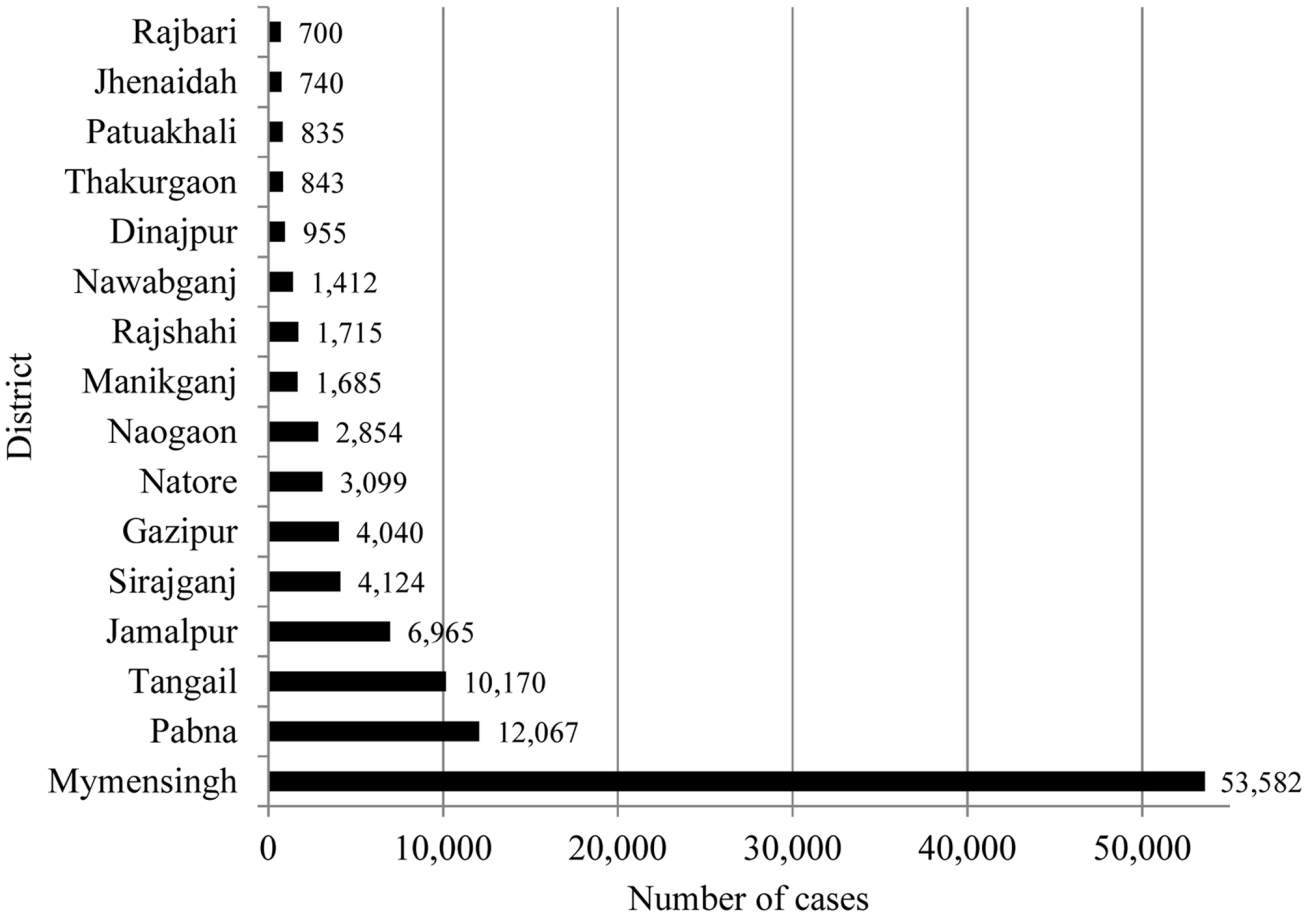
The 16 districts reporting VL cases consistently from 1994 to 2013. (Source: Malaria and Vector-Borne Disease Control Unit, Directorate General of Health Services, Government of Bangladesh, Dhaka).

**Figure 3:**
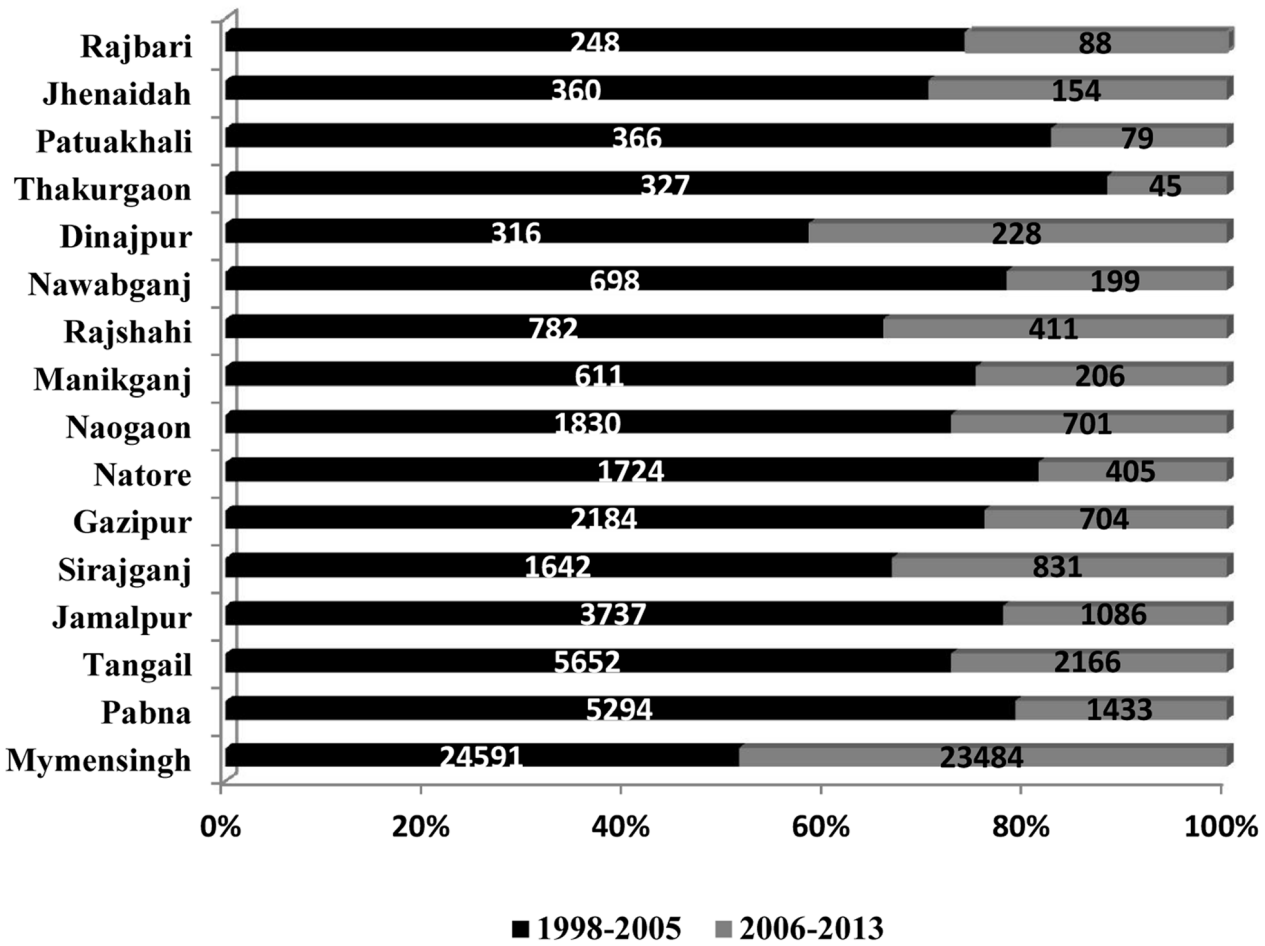
Comparison of the percentage-wise share in the total number of VL cases per district for the period of eight years before (1998–2005) and after (2006–2013) signing the memorandum of understanding by the Health Ministers from Bangladesh, India, and Nepal to eliminate VL from their respective countries by 2015. Source: Malaria and Vector-Borne Disease Control Unit, Directorate General of Health Services (DGHS), Dhaka, Government of Bangladesh.

**Figure 4:**
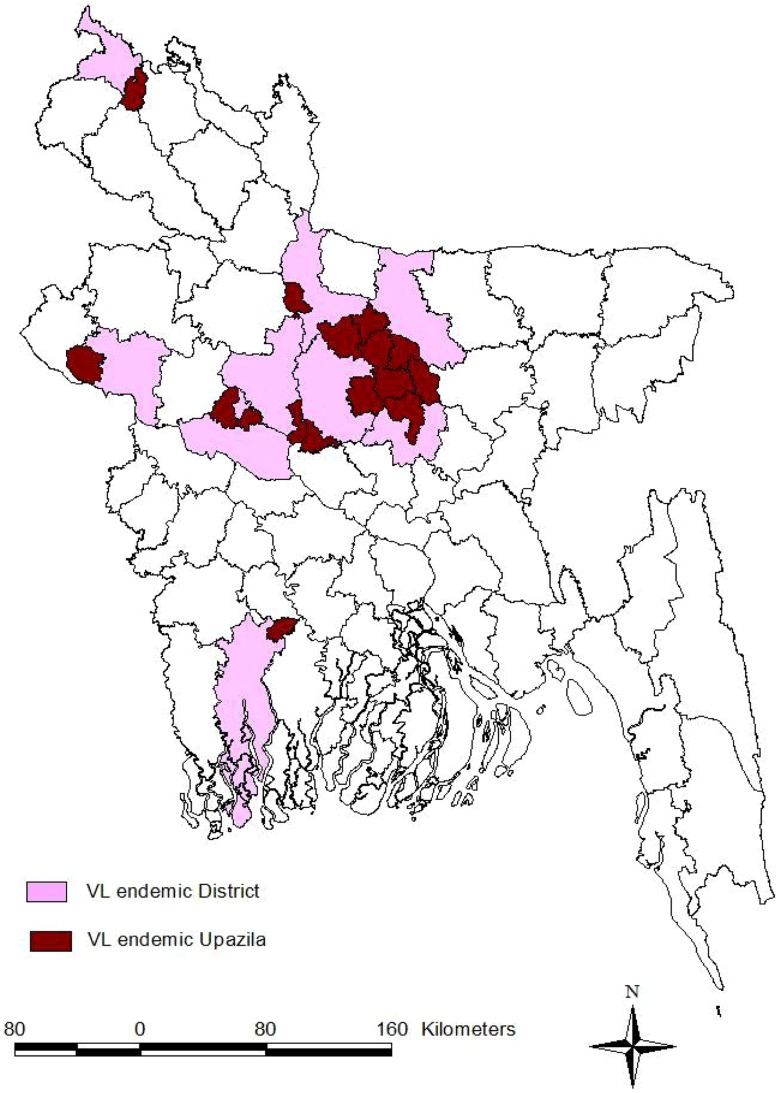
VL-endemic upazilas above target, where incidence rate is above one per 10,000 people from 2008 to 2013, with their respective districts.

From 1994 to 2013, the DGHS of Bangladesh reported 109,266 cases of VL and 329 VL-related deaths from 37 endemic districts. During this 20-year period, there were three years (1997, 2002, and 2006) with more than 8,000 reported cases. The highest number of cases was reported in 2006, and the annual number of cases diminished after that peak year (Figure 1). Altogether, 37 districts reported VL cases at some point during this period. The number of districts reporting VL cases increased from 21 districts in 1994 to 29 districts in 1998 and 2008. Sixteen districts reported VL cases continuously throughout the 1994–2013 period and accounted for 96.82% of the total case number reported during this period (Figure 2). The highest number of VL cases was reported from the Mymensingh district, accounting for 49.04% (53,582) of the total number of cases (Figure 2). The Mymensingh district reported 110 deaths due to VL, or 33.85% of the country's total. The second highest number of cases occurred in the Pabna district (12,067 or 11.04%) (Figure 2) with 11 deaths, followed by the Tangail and Jamalpur districts where 10,170 (9.31%) and 6,965 (6.37%) cases, respectively, were reported (Figure 2), including 45 (13.85%) and eight (2.46%) deaths. Twelve districts reported more than 1,000 cases in the study period, six between 500 and 999, and 19 reported between one and 499 cases. Figure 3 compares the VL case load in the country for the period of eight years before (1998–2005) and after (2006–2013) the signing of the MoU, and shows that except the Mymensingh district, all other endemic districts experienced a remarkable decline of cases. Figure 4 shows 16 upazilas located in nine districts where the average IR ranged between 1.06 to 18.25 per 10,000 population from 2008 to 2013.

The most affected upazilas are situated in the Dhaka division, reporting the highest number of cases of all the divisions (78,079, including 25 deaths from 1994 to 2013). Table 1 shows that cases were reported from 101, 81, 84, 75, 76, and 62 upazilas out of 130, 134, and 140 upazilas at risk, respectively, from 2008 to 2013. The most affected division, Dhaka, reported 4,226, 3,679, 2,115, 3,273, 1,464, and 1,094 cases from 2008 to 2013, respectively, followed by the Rajshahi division, with 573, 530, 604, 584, 359, and 272 cases. From 2008 to 2013, the Khulna division reported 37, 82, 157, 82, 46, and 49 cases, respectively. The Barishal division reported 84 cases from 2008 to 2013, and the Chittagong division reported a very low number of cases (Table 1).

**Table 1:** Cases and deaths reported from 37 endemic districts under five divisions showing the number of VL-endemic upazilas as well as the number reporting cases that year.

Division	District	2008			2009			2010			2011			2012			2013			Deaths (2008–2013)
		Upazila		Cases	Upazila		Cases	Upazila		Cases	Upazila		Cases	Upazila		Cases	Upazila		Cases	
		Endemic	Reporting		Endemic	Reporting		Endemic	Reporting		Endemic	Reporting		Endemic	Reporting		Endemic	Reporting		
Rajshahi	13	74	57	573	74	39	530	76	43	604	76	38	548	76	36	359	76	32	272	6
Khulna	5	8	4	37	8	5	82	8	6	157	8	4	82	8	4	46	8	3	49	2
Barishal	3	2	1	2	3	2	2	3	2	3	3	2	31	3	2	33	3	2	13	0
Dhaka	14	44	38	4,226	47	35	3,679	51	33	2,115	51	31	3,273	51	34	1,464	51	27	1,094	27
Chittagong	2	2	1	2	2	0	0	2	0	0	2	0	0	2	0	0	2	0	0	0
**Total**	**37**	**130**	**101**	**4,840**	**134**	**81**	**4,293**	**140**	**84**	**2,879**	**140**	**75**	**3,934**	**140**	**76**	**1,902**	**140**	**62**	**1,428**	**35**

Source: Malaria and Vector-Borne Disease Control Unit, Directorate General of Health Services, Government of Bangladesh, Dhaka.

Table 2 shows that Mymensingh is the most affected district. The Incidence Rate (IR) per 10,000 people per year varies from 0.1 to 16.5 in the seven affected upazilas (Table 3). As Table 3 shows, almost all cases are concentrated in five of the seven upazilas. The Tangail district has three endemic upazilas, there are two affected upazilas in the Pabna district, and the other six districts have six endemic upazilas (one in each) (Table 2). Among the five most endemic upazilas in the Mymensingh district, the Fulbaria upazila diagnosed 4,085 cases from 2008 to 2013. The second highest case number diagnosed is in the Trishal upazila, where 4,020 cases were diagnosed, followed by Bhaluka (1,501), Muktagacha (1,310), Gafargaon (1,053 cases), Haluaghat (120 cases), and Nandail (21 cases). Other upazilas diagnosed few cases from 2008 to 2013 (Table 3). The IR in the five highly endemic upazilas, Trishal, Fulbaria, Muktagacha, Gafargaon, and Bhaluka was 16.5, 15.7, 5.5, 5.2, and 4.5 per 10,000, respectively, from 2008 to 2013 (Table 3).

**Table 2:** Number of VL cases and annual Incidence Rate (IR) per 10,000 in upazilas.

District	Upazila	2008	2009	2010	2011	2012	2013	Average IR
Mymensingh	Trishal	1,492	1,279	735	564	252	235	
		36.54	30.91	17.53	13.27	5.85	5.38	18.25
Mymensingh	Fulbaria	1,315	781	456	1,608	397	211	
		30.25	17.73	10.22	35.55	8.66	4.54	17.82
Mymensingh	Bhaluka	235	285	125	107	86	73	
		6.93	8.29	3.59	3.03	2.40	2.01	4.35
Mymensingh	Muktagacha	198	343	214	99	82	127	
		4.93	8.42	5.19	2.37	1.94	2.96	4.30
Mymensingh	Gafargaon	260	240	260	292	241	82	
		4.62	4.21	4.50	4.98	4.06	1.36	3.95
Jamalpur	Madarganj	115	109	47	88	64	35	
		4.50	4.21	1.79	3.31	2.37	1.28	2.91
Khulna	Terokhada	46	43	39	17	16	21	
		3.81	3.51	3.15	1.35	1.26	1.63	2.45
Tangail	Shakhipur	70	58	103	73	25	32	
		2.64	2.15	3.78	2.64	0.89	1.13	2.20
Panchagar	Debiganj	18	49	44	96	38	16	
		0.88	2.37	2.10	4.52	1.76	0.73	2.06
Rajshahi	Godagari	70	102	14	47	17	24	
		2.29	3.29	0.45	1.47	0.53	0.73	1.46
Pabna	Faridpur	23	23	19	18	20	17	
		1.69	1.67	1.36	1.27	1.39	1.17	1.42
Sirajganj	Chauhali	26	30	19	20	26	23	
		1.53	1.74	1.09	1.13	1.45	1.26	1.37
Tangail	Nagorpur	39	47	25	39	38	37	
		1.37	1.63	0.86	1.32	1.27	1.22	1.28
Gazipur	Shreepur	55	53	42	58	15	49	1.19
		1.49	1.41	1.11	1.51	0.38	1.29	
Tangail	Modhupur	80	45	55	50	48	49	1.14
		1.72	0.96	1.15	1.03	0.98	0.99	
Pabna	Chatmohor	27	36	36	31	30	13	
		1.02	1.35	1.33	1.13	1.08	0.46	1.06

Approximate number of population was determined based on national growth rate of 1.34 from 2001 to 2013 and Incidence Rate (IR) was calculated per 10,000 people.

Note: in the table first value = reported cases, second value = IR.

Source: Malaria and Vector-Borne Disease Control Unit, Directorate General of Health Services, Government of Bangladesh, Dhaka.

**Table 3:** Number of kala-azar cases and deaths reported by the Mymensingh district from 2008 to 2013.

	Number Diagnosed cases	Total (%)	Number Treated cases	Total (%)	Incidence rate per 10,000 people	Deaths	Case Fatality Rate
**District:**							
Mymensingh district, all upazilas	12,118	100.0	12,089	100.0	4.0	24	0.20
Mymensingh district, 5 highest endemic upazilas	11,969	98.8	11,943	98.8	9.3	23	0.19
**Upazila:**							
Fulbaria	4,085	33.7	4,045	33.5	15.7	3	0.07
Trishal	4,020	33.2	4,284	35.4	16.5	8	0.20
Bhaluka	1,501	12.4	1,526	12.6	4.5	6	0.40
Muktagacha	1,310	10.8	1,325	11.0	5.5	2	0.15
Gafargaon	1,053	8.7	763	6.3	5.2	4	0.38
Haluaghat	120	1.0	117	1.0	0.7	1	0.83
Nandail	21	0.2	21	0.2	0.1	0	0.00
Dobawra	4	0.0	4	0.0	0.0	0	0.00
Ishwarganj	2	0.0	2	0.0	0.0	0	0.00
Phulpur	1	0.0	1	0.0	0.0	0	0.00
Sadar	1	0.0	1	0.0	0.0	0	0.00
Gauripur	0	0.0	0	0.0	0.0	0	-

(Source: Office of the Civil Surgeon, Mymensingh District).

Note: Mean number of kala-azar cases reported per 10,000 per year, based on mean number of estimated district population from 2008 to 2013.

From 2008 to 2013, there were 16 upazilas in nine districts with an average IR exceeding the elimination target, ranging between 1.06 to 18.25 per 10,000 inhabitants (Table 2).

From 2011 to 2013 there were 409, 325, and 240 post kala-azar dermal leishmaniasis (PKDL) cases reported from the Mymensingh district. No country-wide PKDL data are available up to 2012, and 325 cases were reported country-wide in 2013.

## Discussion

The VL elimination program was launched in 2005, and its target was set to reduce the number of cases at upazila level below one case per 10,000 people by 2015 [9]. However, as the initial situation in 2005 and the current epidemiological situation are not well captured, it is extremely difficult to say in 2014 how far we are from the elimination target. Surveillance data from the Disease Control Department of DGHS show that cases were reported from 37 districts from 1994 to 2013, whereas previously, 45 districts were considered endemic [10]. Unfortunately, upazila-level data on VL are not available at DGHS before 2007, making it difficult to properly assess the trends at this level. Moreover, the level of underestimation of VL cases has probably reduced significantly during recent years, as was observed also in India, for at least two reasons: (1) the introduction of a rapid diagnostic test, which has improved access to diagnosis, and (2) the recent policy making free VL treatment available in the public services with drugs that are not available in the private market. This changing, under-reporting ratio does not facilitate the interpretation of trends.

About half of the total numbers of VL cases were reported from a single district (Mymensingh), where five upazilas are highly endemic. Therefore, the national program should intensify its efforts in these areas with high priority. Due to VL, there is a huge economic loss for the affected families in an endemic community [11]. Currently, the access to diagnosis and treatment for VL is improved, as VL care is available free of charge at the UHC or district-level hospital in all endemic districts, which is clear progress. However, to reduce the VL incidence rates, a complete package of activities (prompt case detection, proper treatment and case management, and effective vector control) should be deployed on an urgent basis. Unfortunately, no vector control activities were carried out between 1999 and 2012 in many areas due to financial constraints, lack of trained human resources, and unavailability of insecticides for indoor residual spraying (IRS) because of procurement problems [12]—except for limited IRS in a few pilot projects in 2011 in the Mymensingh district. In 2012, IRS was implemented in eight highly endemic upazilas (Fulbaria, Trishal, Bhaluka, Gaforgaon, and Muktagacha in Mymensingh, Terokhada in Khulna, Madarganj in Jamalpur, and Nagorpur in Tangail) during the pre- and post-monsoon periods. Focal spraying was conducted in moderate- and low-endemic areas from January 2013. Recently insecticide-treated bed nets were distributed to all patients treated in UHCs in the highly endemic communities in the past three years.

In 2011, the Fulbaria upazila of the Mymensingh district reported 1,608 cases. The most likely explanation for this high figure is that in mid-2010 an international NGO (MSF) started a VL control program in this upazila through active case search and treatment with AmBisome. They also provided food during hospitalization, and compensation for transport and wage losses. This intervention attracted a high number of patients, including some living in nearby upazilas, who used the address of their relatives or friends in Fulbaria as their own address in order to get access to the best facilities.

Passive surveillance records show 329 patients died in 20 years (1994–2013), which is probably an under-estimation, as there was drug scarcity before the introduction of Miltefosine as a first-line drug. Poor quality SSG was used during periods of drug scarcity, and there was a major incidence of the use of substandard Miltefosine for a certain period, which added to mortality [13]. The data on VL deaths were mostly recorded in patients who died in the government hospitals during drug administration, but those who died at home or in a private clinic due to VL or its complications were not recorded by passive surveillance. A community-based study showed that the VL case fatality rate in Bangladesh was 5.3% in males and about three times higher in females [14].

The reporting of PKDL cases should be strengthened, as country-wide data are lacking up to 2013. A recent study from Bangladesh shows that the cumulative incidence of PKDL can be up to 17% within five years of being treated for kala-azar [15]. Another study, performed in a less VL-endemic area, found a PKDL prevalence of about 6 per 10,000 people [16]. These PKDL cases are important for transmission dynamics as they are supposedly highly infectious.

Effective progress towards VL elimination requires continuous surveillance (Box 1) of mortality and morbidity as well as of the populations at risk. While clear progress has been made towards VL elimination in Bangladesh, 16 of 140 endemic upazilas had not yet reached the target in 2013, based on official notification data that suffer from underreporting bias. The elimination initiative urgently needs to establish methods to ascertain and monitor the elimination target.

**Box 1. Importance of Proper Disease Surveillance for VL Elimination Program**

- Measure the past and present disease burden.
- Guide national program to take timely and appropriate action on:

○Patient management

○Vector control

○Community awareness through IEC

- Prevent unnecessary program expenditure, i.e., accurate surveillance record will provide guidance on specific areas in which to take necessary actions instead of in the whole upazila or union.
- Predict future burden.

**Key Learning Points**

- Human resources responsible for epidemiological surveillance, including data management, require intensive training and supervision.
- Effective surveillance is crucial to understand the real burden of disease and for taking timely action.
- It is essential to have a proper referral system for the VL patients so as to avoid the duplication of reporting.
- Community-level health staff needs training to identify the cause of death.
- The private sector clinics and practitioners should be involved in the reporting system for VL.

**Top Five Papers**

1. Bern C, Chowdhury R (2006) The epidemiology of visceral leishmaniasis in Bangladesh and prospects for improved control. Indian J Med Res 123: 275–288.
2. Mondal D, Alam MS, Karim Z, Hague R, Boelaert M, et al. (2008) Present situation of vector control in Bangladesh: a wake up call. Health Policy 87: 369–376.
3. Ahluwalia IB, Bern C, Costa C, Akter T, Chowdhury R, et al. (2003) Visceral Leishmaniasis: Consequences of neglected disease in a Bangladeshi community. Am J Trop Med Hyg 69: 624–628.
4. Ahluwalia IB, Bern C, Wagatsuma Y, Costa C, Chowdhury R, et al. (2004) Visceral Leishmaniasis: Consequences to Women in a Bangladeshi Community. J Womens Health (Larchmt) 13: 360–364.
5. Anoopa Sharma D, Bern C, Varghese B, Chowdhury R, Haque R, et al. (2006) The economic impact of visceral leishmaniasis on households in Bangladesh. Trop Med Int Health 11: 757–764.
